# The Dutch clinical impairment assessment: factor analysis and psychometric properties in a clinical eating disorder sample

**DOI:** 10.1007/s40519-025-01767-8

**Published:** 2025-07-21

**Authors:** Daniela Schlochtermeier, Matthijs Blankers, Jaap Peen, Elske van den Berg, Ella van Beers, Bernou Melisse, Jitske Koenders, Anna E. Goudriaan, Margo de Jonge, Jack Dekker, Edwin de Beurs

**Affiliations:** 1 Novarum Center for Eating Disorders & Obesity, Amstelveen, The Netherlands; 2 Arkin Mental Health Institute, Amsterdam, The Netherlands; 3 Trimbos Institute, Amsterdam, Utrecht, The Netherlands; 4 Department of Clinical Psychology, Leiden University, Leiden, The Netherlands; 5 American Center for Psychiatry and Neurology, Abu Dhabi, United Arab Emirates; 6 Co-Eur, Utrecht, The Netherlands; 7 Mentrum Mental Health Care, Amsterdam, The Netherlands; 8 Amsterdam UMC, University of Amsterdam, Amsterdam, The Netherlands; 9 Faculty of Behavioural and Movement Sciences, Vrije University, Amsterdam, The Netherlands

**Keywords:** Clinical impairment assessment, Bifactor analysis, Eating disorders, Psychometrics

## Abstract

**Purpose:**

The present study reports on the psychometric properties of the Dutch translation of the clinical impairment assessment (CIA) questionnaire in female patients with eating disorders. The aim of this study was to determine the factor structure of the CIA as there are conflicting studies supporting a three-factor, bifactor, and single-factor model with a general factor and three specific factors.

**Methods:**

The CIA was translated and administered to 321 female patients with various eating disorders receiving treatment in a specialized eating disorder center. Its factor structure, internal consistency, convergent validity, and sensitivity to change were investigated.

**Results:**

Confirmatory factor analyses showed the best fit was a bifactor model with one strong general factor and three less strong specific factors for personal, social, and cognitive impairment. Furthermore, good internal consistency (Cronbach’s *α* = 0.91), good convergent validity between CIA global score and eating disorder examination questionnaire global score (*r* = 0.58; *p* < 0.001) and good sensitivity to change (*t* (115) = 13.76, *p* < 0.001) were found.

**Conclusions:**

The Dutch CIA is a reliable and valid instrument to measure impairment secondary to eating disorder symptoms, but interpretations made from subscales scores should be used with caution.

**Level of evidence:**

Level III, validation study.

## Introduction

Eating disorders are marked by disruptive and maladaptive eating behaviors, such as restrictive eating, binge eating, purging, over-exercising, along with the core psychopathology of overevaluation of shape and weight which can have a negative effect on one’s physical and mental health [1]. Secondary to the eating disorder symptoms and core psychopathology, eating disorders are known to impair a patient’s functioning in multiple domains of life, including their professional, academic, personal, and social life and lead to a decrease in quality of life [2]. This impairment is one of the reasons that patients seek help; therefore, treatment should also aim to ameliorate this impairment [3]. Functional impairment is important to research as a treatment outcome as it is not only important for the patient, but it is also an important diagnostic criterion across all mental illnesses, including eating disorders [4].

Four quality-of-life questionnaires have been developed specifically for eating disorders: the Quality of Life for Eating Disorders questionnaire [5], the Eating Disorder Quality of Life Scale [6], and the Health-Related Quality of Life in Eating Disorders Questionnaire, a revised version of the Spanish Health-Related Quality of Life in Eating Disorders Questionnaire [7], and the Eating Disorders Quality of Life Instrument [8]. Only the Eating Disorders Quality of Life Instrument takes patients’ evaluation of eating and weight into consideration which the others do not and none of the above-mentioned scales measure the core psychopathology as defined by overevaluation of weight and shape. The Eating Disorders Quality of Life Instrument [8] specifically refers to impairment due to eating and weight but not due to a patient’s concerns and cognitions about their shape and weight, and therefore misses an important aspect of the impact of the core psychopathology.

To address the concern that previously developed instruments do not fully take the impact of the overevaluation of shape and weight into consideration, Bohn et al. [3] developed the 16-item clinical impairment assessment (CIA). The CIA assesses the psychosocial impairment suffered by patients as a direct result of their eating- and exercise habits, as well as the consequences of the core psychopathology of eating disorders. In the original study, the CIA showed good psychometric properties including internal consistency, construct and discriminant validity, test–retest reliability, and a high sensitivity to change [3].

Replication studies of the original CIA questionnaire across patients with eating disorders in clinical and community samples supported its reliability and validity in the United Kingdom [9] and the United States [10]. Translated versions of the CIA are reliable and valid for measuring secondary impairment due to core psychopathology of eating disorders in Norway [11], Fiji [12], Spain [13], Italy [14], and Portugal [15].

There is a gap in the literature regarding the factor structure underlying the CIA, as this is an essential element of construct validity, and it impacts how the instrument should be used and scored. The three-factor model that was initially proposed is supported by confirmatory factor analyses in Spain [13], Italy [14], Portugal [15], and the United Kingdom [9]. However, in early stages of developing this instrument, Bohn [3] proposed measuring “impairment overall and in three specific domains (personal, cognitive, social).” (p. 1105), which applies to a bifactor model with each item loading on a general factor (general impairment) and its purported factor. Therefore, the overall factor was computed by averaging all items and computing the subscale scores independently. However, Raykos et al. [16] were the first to investigate different factor models and found the best fit for the bifactor model. These findings were replicated by Maraldo et al. [17], whereby the bifactor model had the best fit in a clinical sample. Raykos and Maraldo both found a reliable general factor, but subscale factors appeared insufficiently reliable and thus recommended not using these subscales.

This study investigated the factor structure of the Dutch version of the CIA and its psychometric properties on female patients. We closely followed the analytic approach of Raykos et al. [16] and evaluated four models: a general factor model, a three-factor model with independent factors, a three-factor model with correlated factors, and a bifactor model. Rodriguez et al. [18] proposed indices and criteria to evaluate whether a bifactor model is supported by the data, quantify the relative importance of the general factor and three other factors, and therefore establish whether a calculation of subscale scores is justified. This involves the inspection of fit indices of the models, examining the reliability coefficient omega, evaluating the ratio of explained variance by the general factor, and explaining the variance by the general factor and three specific factors. We hypothesized a superior fit of a bifactor model over a unidimensional model or a three-factor model. Next to analyzing the factor structure, this study investigated additional psychometric properties of the Dutch translation of the CIA, including internal consistency, reliability, convergent validity, and responsiveness, which we expect to be sufficient after translation.

## Methods

### Participants and procedure

The study sample consisted of female inpatients and outpatients receiving care at a specialized eating disorder treatment center, who were referred between 2015 and 2017. All patients were included, with the exclusion of patients with avoidant/restrictive food intake disorder or a body mass index > 40. This sample was part of a naturalistic study examining the effectiveness of CBT-E (for further information on this study and its exclusion criteria, see Van den Berg et al. [19]). Clinical psychologists or psychiatrists diagnosed patients according to the Diagnostic and Statistical Manual, criteria DSM-IV [20] or DSM-5 criteria [1] for an eating disorder. In addition to their diagnostic assessment, all patients completed the Eating Disorder Examination-Questionnaire (EDE-Q) [21, 22], the Outcome Questionnaire 45 [23–25] and the CIA. Assessments were conducted at intake and at the end of treatment.

### Measures

The CIA is a 16-item self-report questionnaire measuring psychological impairment secondary to eating disorder symptoms over the past 28 days using three subscales: personal (e.g., ….made you more critical of yourself), social (e.g., … stopped you going out with others), and cognitive (e.g., …made it difficult to concentrate). Items are scored on a 4-point Likert scale (0 = not at all, 3 = a lot), and the global score is the sum of all individual scores, provided that a minimum of 12 items are answered. The cut-off score is 16 and differentiates between having an eating disorder and not having an eating disorder (UK norms). Earlier studies replicated the original findings of good psychometric properties including high internal consistency, sensitivity to change, construct, and discriminant validity [3, 11, 13]. The CIA was first translated from English to Dutch by a bilingual researcher, and then backtranslated into English by a second bilingual researcher. Both versions were compared, necessitating minor changes before reaching consensus on the accuracy of the Dutch version by investigators.

This study used the Dutch version of the EDE-Q [22], a 36-item self-report questionnaire consisting of four subscales: dietary restraint, eating concern, shape concern, and weight concern. The past 28 days are covered, and items are scored on a 7-point Likert scale. Scores per subscale are the average score of all items in the scale, and the global score is the average score of all items. A cut-off score for remission is used and defined as a global EDE-Q score lower than one standard deviation above the community means, or below 2.77 based on United Kingdom norms for comparison [21].

The Dutch version of the Outcome Questionnaire-45 is a 45-item self-report questionnaire yielding a global score and three subscale scores for symptomatic distress, interpersonal relations, and social role [24]. The OQ-45 is scored on a 5-point frequency scale (0 = never, 4 = almost always) and shows adequate psychometric properties with a cut-off score of 57 [24].

### Statistics

To examine the factor structure of the CIA, the data-analytic approach of Raykos et al. [16] who applied the guidelines of Rodriguez et al. [18] was followed. Multidimensionality of the CIA was tested with confirmatory factor analysis with the “lavaan” package on R [26]. As scores on most items were not normally distributed, the estimation algorithm for ordinal data (diagonally weighted least squares (DWLS) and present scaled indices were used. The following goodness of fit indices used for the confirmatory factor analysis were comparative fit index (CFI) and Tucker–Lewis Index (TLI). For both indices, a value above 0.9 was considered indicative of a good fit. The root mean square error of approximation (RMSEA) indicated a good fit if below 0.06 and the standardized root mean residuals (SRMR) were a good fit if below 0.08 [27].

Several bifactor indices were used. Explained common variance (ECV) used the variance explained by the general factor, divided by the variance explained by the general and group factors. The percentage of uncontaminated correlations (PUC) used the percentage of covariance terms which only reflect variance from the general dimension. The relative parameter bias (RPB) used the difference between an item loading in the unidimensional solution and its general factor loading in the bifactor solution. The average RPB (ARPB) used the average of the item RPB’s. An ARPB lower than 0.10–0.15 is acceptable [18, 28]*. H* is a measure of construct replicability, with an *H* > 0.80 suggesting a well-defined latent variable and H < 0.70 suggesting a poorly defined latent variable [18]. Indices were calculated using the Bifactor Indices Calculator on Microsoft Excel [29].

To assess internal consistency, Cronbach’s alpha and McDonald’s omega coefficient were used. Cronbach’s alpha was used to compare the present findings with other validation studies. Convergent validity was assessed by using Spearman’s correlation between global scores of the CIA, EDE-Q and OQ-45 scores.

In line with the original study, the sensitivity to change was assessed by calculating whether there was a significant decrease on the global CIA score between the first assessment and the end-of-treatment assessment using a paired sample *t*-test. Effect sizes were calculated using Cohen’s *d* (small, medium, and large, respectively, >0.20, >0.50, and >0.80). Sensitivity to change of the CIA global was compared to the EDE-Q global score with multivariate repeated measures analyses in a time-by-instrument factorial design. A significant interaction of time by instrument would show differential responsiveness of both measures. These analyses were done using SPSS, version 25.

## Results

Sample characteristics are presented in Table 1. The mean global score at pretest on the CIA was 30.77 (SD = 9.67). Participants scored above the cut-off score on the CIA (*n* = 293, 91.3%), EDE-Q (*n* = 234, 72.9%) and OQ-45 (*n* = 256, 79.8%).

**Table 1:** Baseline characteristics of *N* = 321 female patients with various eating disorders

Age [mean (SD); range]	27.9 (8.96); (18–68)
Body mass index [mean (SD); range]	21.8 (5.2); (12.7–39.9)
Diagnosis *n*, (%)	
Anorexia nervosa	90 (28%)
Bulimia nervosa	100 (31%)
Binge-eating disorder	31 (10%)
Otherwise-specified feeding or eating disorder	100 (31%)
CIA [mean (SD); range]	30.77 (9.67); (1–48)

### Factor analyses

Robust fit statistics are presented in Table 2. The unidimensional model and the model with three independent factors had an insufficient fit. Allowing correlation among the factors yielded to a model with an improved fit, but only the bifactor model had a sufficient fit across all indices. Given the bifactor model had the best fit, we assessed the relative contribution in variance by the general and group factors (Table 2) using the indicators recommended by Rodriguez et al. [19].

**Table 2:** Robust fit indices for confirmatory factor analysis models

Model	χ^2^	*df*	CFI	TLI	RMSEA	90% *CI*		SRMR
						*LL*	*UL*	
Unidimensional	773.6***	104	**0.955**	**0.948**	0.142	0.133	0.151	0.115
Three independent factors	6011.6***	104	0.605	0.544	0.421	0.412	0.430	0.346
Three correlated factors	242.2***	101	**0.991**	**0.989**	0.066	**0.055**	0.077	**0.063**
Bifactor	115.9*	88	**0.998**	**0.997**	**0.031**	**0.012**	**0.046**	**0.048**
Good fit guidelines			>0.95	>0.95	<0.06			<0.08

***p < 0.001; *p < 0.05

*CFI* comparative fit index, *TLI* Tucker–Lewis index, *RMSEA* root mean error of approximation, *SRMR* standardized root mean residuals

*LL* and *UL* lower and upper limits of the 90% confidence interval. Sufficient fit is indicated in bold typeface

The internal consistency of the CIA global score was excellent (Cronbach’s *α* = 0.91), and the subscales also showed good internal consistency (≥0.84). The Omega reliability coefficients for the CIA global score (0.94) and each subscale (≥0.86) were very high. The omega-hierarchical value was also high (0.82), whereas the Omega hierarchical-subscale values were quite low (≤0.39), and the coefficient H-values (≤0.73) of the subscales were below the 0.80 threshold. Coefficient H-values of the global score (0.93) were above the threshold. These indices of the bifactor model are displayed in Table 3.

**Table 3:** Bifactor indices of the CIA among eating disorder patients

	General	Personal	Social	Cognitive
Omega	0.94	0.88	0.86	0.88
Omega H/Omega HS	0.82	0.39	0.08	0.37
ECV	0.66	0.45	0.22	0.34
PUC	0.71			
ARPB	0.14			
H	0.93	0.73	0.53	0.69
Cronbach’s *α*	0.91	0.88	0.84	0.86

*Omega* Omega total/Omega subscale, *ECV* explained common variance, *PUC* percentage of uncontaminated correlations, *ARPB* average relative parameter bias, *H* coefficient H construct reliability

Table 4 presents factor loadings for the unidimensional and bifactor models. Loadings for the unidimensional model are presented for comparison. Loadings were medium to high on the general factor. Factor loadings on the subscales were acceptable, apart from items 3, 12, and 15, all belonging to the social subscale, as these loadings were very low.

**Table 4:** Standardized factor loadings for unidimensional and bifactor confirmatory factor analysis solutions in the clinical sample

Item		Uni	General	Personal	Social	Cognitive
1	… made it difficult to concentrate?	0.75	0.74			0.30
2	… made you more critical of yourself?	0.67	0.58	0.45		
3	… stopped you going out with others?	0.72	0.74		0.22	
4	… affected your performance at work (if applicable)?	0.67	0.66			0.26
5	… made you forgetful?	0.75	0.58			0.78
6	… affected your ability to make everyday decisions?	0.74	0.71			0.34
7	… interfered with meals with family or friends?	0.72	0.67		0.57	
8	… made you upset?	0.78	0.62	0.60		
9	… made you feel ashamed about yourself?	0.79	0.60	0.66		
10	… made it difficult to eat out with others?	0.71	0.65		0.61	
11	… made you feel guilty?	0.72	0.58	0.55		
12	… interfered with your doing things you used to enjoy?	0.74	0.81		0.01	
13	… made you absent-minded?	0.75	0.64			0.51
14	… made you feel a failure?	0.74	0.63	0.52		
15	… interfered with your relationship with others?	0.71	0.78		0.03	
16	… made you worry?	0.66	0.57	0.46		

*Uni* unidimensional model loading

Convergent validity: At pretest, the Spearman Rho correlation between the CIA and the other measures was statistically significant and substantial (with EDE-Q *r* = 0.58; *p* < 0.001; *n* = 267); with OQ-45 *r* = 0.58; *p* < 0.001; *n* = 260). At the post-test, correlation coefficients also suggested strong convergence (CIA with EDE-Q *r* = 0.53; *p* < 0.001; *n* = 107); with OQ-45 *r* = 0.54; *p* < 0.001; *n* = 96).

Sensitivity to change: There was a significant decrease in the global CIA score between admittance *M* = 31.27 (SD = 9.46) and the end of the treatment *M* = 17.37 (SD = 12.07), *t* (115) = 13.76, *p* < 0.001,* d* = 1.28). The repeated measure MANOVA showed a significant time effect, no instrument effect and only a marginal time by instrument effect *F*(1,106) = 3.82, *p* = 0.053, *partial eta2* = 1.81. There was a non-significant difference pre/post measure between the EDE-Q total score and CIA total score with a slightly higher standardized difference for EDE-Q 1.81 than CIA 1.33, respectively.

## Discussion

The present study aimed to examine several psychometric properties of the Dutch CIA, including its factor structure. Findings indicated that the bifactor model had the best fit compared to the other models tested. The psychometric properties were good with high internal consistency, good sensitivity to change, and good convergent validity.

The findings of the factor structure are in line with the findings by Raykos et al. [16] and Maraldo et al. [17]. The bifactor structure had the best fit with a strong general factor explaining most of the variance and less reliable subscales. In contrast to Raykos et al. [16], these findings suggest that a reasonable amount of variance can be explained by the subscales, particularly the personal subscale and to some degree, the cognitive subscale. Results showed acceptable loadings on the general factor and personal subscales. For the social subscale, acceptable loadings for only two of the five items were found. For the cognitive subscale, acceptable loadings were found for two out of six of the items that were expected to predominantly load on this scale. The findings for the bifactor structure revealed that the CIA measures a general factor and that of the three subscales, only the impairment in personal functioning subscale can reliably be interpreted independent of the general factor. These findings indicate that the CIA is a reliable measure of the general impairment factor. However, the subscales are measured in the same construct, and are therefore less reliable. Given that both omega-HS and omega-H are very low on the cognitive and social scales, these factors are not reliably measured by these scales. Therefore, the factor structure is likely to vary across studies [18]. The problematic items that were found in the present study were also reported by Maraldo et al. [17] and Raykos et al. [16]. Furthermore, all studies found convergent results on the low loading items.

The instrument’s general score is reliable and valid, aligning with prior findings in this area. However, the subscale scores should be interpreted with caution; the underperformance of the subscales for social and cognitive functioning do not likely result from a problematic translation of the items or cultural differences, given Raykos et al. [16] and Maraldo et al. [17] both used the original version and reported similar findings. To enhance reliability and usability of the CIA subscales, future research should aim at editing or rewriting these items or extending the subscales with additional items to improve the factor structure of the instrument.

Regarding the other psychometric properties of the translated version, the CIA possesses sufficient internal consistency, sufficient reliability and appears to be a valid measure for assessing secondary impairment due to eating disorder symptoms. All items contributed to the global score of the CIA, with both the Cronbach’s *α* and Omega coefficient demonstrating good internal consistency across the three subscales. Together these findings align with other CIA validation studies [9–14, 30, 31]. The scale had good convergent validity, with patients with more severe eating disorder psychopathology (EDE-Q) and higher general psychopathology (OQ-45) reporting higher impairment on the CIA. This was the case both at the beginning of treatment and end of treatment. These findings are also in line with findings reported by Vaz et al. [15]. Finally, the sensitivity to change is in line with the original findings by Bohn et al. [3]. As there is a significant decrease between mean CIA global scores at the beginning compared to the end of treatment, the CIA demonstrated good sensitivity to change with a very large effect size. The same results were found for the scores on the EDE-Q with an interaction effect on both instruments. However, the instruments measure different constructs, and the changes could occur at different times during the treatment. Compared to the study by Bohn et al. [3] the CIA mean global score at the end of treatment in our sample was higher, perhaps due to a higher ratio of patients with anorexia nervosa, making successful treatment more difficult.

The CIA general factor, and to some extent the personal subscale, are very useful for practitioners in monitoring change of impairment over the course of treatment and learn about patients’ attitude towards consequences of their ED. It can also be used to motivate change and provide a more thorough understanding of improvements in ED symptoms, as clinical impairment may take more time to restore compared to other ED symptoms such as decreases in strict eating rules.

### Strengths and limitations

This study has several strengths. It is the first study to investigate the facture structure and psychometric properties of the Dutch version of the CIA. Furthermore, this study investigated a large sample of inpatients and outpatients who all suffered from severe eating disorders. Another strength is that, to our knowledge, this study investigated the largest group of patients with binge-eating disorder thus far to examine the psychometric properties of the CIA.

A limitation of this study is the lack of male participants. Therefore, the present finding only apply to women. Another limitation is a lack of CIA data from a community sample, since the used data were part of a clinical study and not solely gathered to validate the CIA. Due to the lack of a community sample no cut-off scores on the Dutch CIA for caseness was investigated and instead the originally recommended cut-off score was used [3]. For further research, we recommend gathering CIA data for a broader range of eating disorder diagnoses, in a community sample, and with more male patients to investigate an optimal clinical cut-off score, as this will enhance the utility and generalizability of the instrument. We would also recommend the rephrasing of items 12 and 15 as convergent findings from this study and prior validation studies suggest that these items do not establish a strong and reliable subscale. Another recommendation is the development of norms for females and males separately to allow for more meaningful interpretation of scores and use in clinical practice.

### What is already known on this subject?

The CIA is a useful and frequently used measure of impairment relating to a psychiatric disorder, however, conflicting reports on its psychometric properties have been reported. The CIA has been widely translated and validated across cultures; however, the existing Dutch version had not been assessed or validated.

### What does this study add?

Based on the finding that the Dutch version of the CIA is reliable and valid, it can be widely used to monitor outcomes for Dutch-speaking patients with eating disorders. This study contributes to the current understanding of assessing and monitoring clinical impairment for those with eating disorders, and these findings validate the use of the CIA in future research and policy.

## Conclusion

In conclusion, as functional impairment is one of the primary reasons for patients with eating disorders to seek help, it is important to have an instrument that can measure the extent of this impairment. Assessing impairment is essential to establish the severity of patients’ conditions at the onset of treatment, as well as monitor their progress over time. With this study, we evaluated the factor structure and other psychometric properties of the Dutch CIA and found that the instrument had a bifactor structure with a reliable general factor and a specific personal factor with sufficient reliability, high internal consistency, good known group validity, sensitivity to change, and convergent validity. 

## Data Availability

No datasets were generated or analyzed during the current study.
